# The effect of a split-dose intravenous dexamethasone and a single high-dose on postoperative blood glucose after total joint arthroplasty: a randomized double-blind placebo-controlled trial

**DOI:** 10.1186/s13018-024-04887-6

**Published:** 2024-07-02

**Authors:** Xiao-yang Liu, Ping Mou, Jian Cao, Xu-ming Chen, Hao-Yang Wang, Wei-Nan Zeng, Zong-Ke Zhou

**Affiliations:** 1 Department of Orthopedics, Orthopaedic Research Institute, West China Hospital, Sichuan University, #37 Guoxue Road, Chengdu, 610041 People’s Republic of China; 2 Department of Rehabilitation Medicine, West China Hospital, Sichuan University, #37 Guoxue Road, Chengdu, 610041 People’s Republic of China; 3 Key Laboratory of Rehabilitation Medicine in Sichuan Province, West China Hospital, Sichuan University, #37 Guoxue Road, Chengdu, 610041 People’s Republic of China

**Keywords:** Total joint arthroplasty, Dexamethasone, Fasting blood glucose, Postprandial blood glucose

## Abstract

**Background:**

In patients undergoing total joint arthroplasty (TJA), the administration of dexamethasone may contribute to perioperative blood glucose (BG) disturbances, potentially resulting in complications, even in patients without diabetes. This study aimed to demonstrate the impact of different administration regimens of dexamethasone in postoperative BG levels.

**Methods:**

In this randomized, controlled, double-blind trial, 136 patients without diabetes scheduled for TJA were randomly assigned to three groups: two perioperative saline injections (Group A, placebo); a single preoperative injection of 20 mg dexamethasone and a postoperative saline injection (Group B), and two perioperative injections of 10 mg dexamethasone (Group C). Primary outcomes were the postoperative fasting blood glucose (FBG) levels. Secondary outcome parameters were the postoperative postprandial blood glucose (PBG) levels. Postoperative complications within 90 days were also recorded. Risk factors for FBG ≥ 140 mg/dl and PBG ≥ 180 mg/dl were investigated.

**Results:**

Compared to Group A, there were transient increases in FBG and PBG on postoperative days (PODs) 0 and 1 in Groups B and C. Statistical differences in FBG and PBG among the three groups were nearly absent from POD 1 onward. Both dexamethasone regimens did not increase the risk for postoperative FBG ≥ 140 mg/dl or PBG ≥ 180 mg/dl. Elevated preoperative HbA1c levels may increase the risk of postoperative FBG ≥ 140 mg/dl or PBG ≥ 180 mg/dl, respectively.

**Conclusion:**

Perioperative intravenous high-dose dexamethasone to patients without diabetes has transient effects on increasing BG levels after TJA. However, no differences were found between the split-dose and single high-dose regimens. The elevated preoperative HbA1c, but not the dexamethasone regimens were the risk factor for FBG ≥ 140 mg/dl and PBG ≥ 180 mg/dl.

**Trial registration:**

Chinese Clinical Trail Registry, ChiCTR2300069473. Registered 17 March 2023, https://www.chictr.org.cn/showproj.html?proj=186760.

## Background

Dexamethasone has been extensively used in perioperative intravenous administration to reduce postoperative pain, opioid consumption, and the occurrence of nausea/vomiting following primary total joint arthroplasty (TJA) [1–5]. Recent studies further substantiated that high-dose dexamethasone yield superior efficacy than low-dose dexamethasone [6, 7]. Additionally, compared to a preoperative single high-dose regimen, perioperative multiple doses of dexamethasone injections may further enhance the therapeutic effects [8–11]. Hence, orthopaedic surgeons preferred to multi-dose or high-dose dexamethasone perioperatively to enhance recovery after TJA.

However, as a glucocorticoid, dexamethasone may cause immunosuppression and metabolic disorder [12]. Among the potential complications associated with dexamethasone, disturbances in glucose metabolism are significant and may lead to postoperative hyperglycemia [13]. According to previous research, postoperative fasting blood glucose (FBG) ≥ 140 mg/dl and postprandial blood glucose (PBG) ≥ 180 mg/dl may induce wound complications and periprosthetic joint infection (PJI), even in patients without diabetes [14–19]. Moreover, the higher the dose of dexamethasone is used, the more likely postoperative hyperglycemia is to occur. And Dhatariya et al. demonstrated that patients without diabetes may exhibit heightened sensitivity to dexamethasone-induced hyperglycemia and result in more pronounced postoperative blood glucose (BG) fluctuations [20]. Therefore, studies should be conducted to discuss the impact of dexamethasone on postoperative BG in patients without diabetes undergoing TJA. There are limited studies demonstrating the impact of dexamethasone on postoperative BG in patients undergoing TJA. Nonetheless, current studies on the association of dexamethasone and BG were retrospective and there is a lack of prospective clinical evidence [21, 22]. Moreover, the timing and dose of dexamethasone injections were inconsistent, which increased the confounding factors [23–25]. Considering the trend leans towards multi-dose or high-dose dexamethasone, the impact of these regimens on BG has not been adequately addressed [8, 9, 11]. Therefore, it is necessary to further clarify the effects of multiple and single high-dose dexamethasone on postoperative BG in patients without diabetes.

This randomized controlled trial aimed to compare the postoperative BG levels in patients without diabetes who received different regimens of dexamethasone (0 mg, 10 + 10 mg, and 20 mg). We hypothesized that neither split-dose nor single high-dose dexamethasone would result in elevated postoperative FBG and PBG. Additionally, we explored the relationship between different doses of intravenous dexamethasone and postoperative BG. We also identified the risk factors associated with FBG ≥ 140 mg/dl or PBG levels ≥ 180 mg/dl.

## Methods

### Trial design

We designed a single-center, double-blind, prospective, randomized, placebo-controlled trial from May 2023 to October 2023. The trial was registered in the International Clinical Trial Registry (ChiCTR2300069473) and was approved by our institutional review board (2023-265) before recruitment. The report was in alignment with the Consolidated Standards of Reporting Trials (CONSORT) 2010 guidelines [26].

### Participants

Patients aged between 18 to 80, planned for unilateral elective primary TJA for end-stage osteoarthritis were assessed for eligibility. Patients were excluded when they had (1) preoperative hyperglycemia due to diabetes mellitus or other diseases; (2) steroids use for underlying diseases; (3) been scheduled for revisions or bilateral procedures; (4) severe liver or kidney failure; (5) inflammatory diseases including rheumatoid arthritis, ankylosing spondylitis, or systemic lupus erythematosus. Written informed consent was obtained from each patient.

### Randomization, blinding and intervention

The recruited patients were randomized into three groups in a 1:1:1 ratio with block sizes of six or nine using a computer-based random integer generator (Research Randomizer, www.randomizer.org) by an external trial collaborator. The results were placed into sequentially numbered, opaque, sealed envelopes which were opened on the day of operation. The trial drug was labeled as “trial medication”, to assure blinding. In Group A, patients received two doses of intravenous normal saline, one administered before anesthesia induction, and the second given 24 h later. Group B patients received one intravenous dose of 20 mg dexamethasone before anesthesia induction and one dose of normal saline 24 h later. Group C patients received 10 mg dexamethasone before anesthesia induction and another 10 mg dexamethasone 24 h later. All participants, investigators, surgeons, and statisticians were blind to the intervention. The anesthesiologists performing the interventions were not blind to the treatment, but did not participate in any outcome assessments. The nurses in charge of intravenous injections 24 h postoperatively were also not blind, but no study group identifiers were presented to the nursing staff thereafter. The researchers were unaware of the randomization and were unblinded after the trial was completed.

### Perioperative management

A standardized clinical protocol was implemented for each patient undergoing TJA [11]. All patients received intravenous tranexamic acid at a dose of 20 mg/kg 10 min before skin incision, and additional intravenous doses of 1 g tranexamic acid were administered 3 and 6 h after surgery, respectively.

The multimodal analgesic standard protocol was initiated upon admission, and all patients were administered diclofenac 75 mg twice daily. Before suturing the wound, all patients received local infiltration analgesia consisting of 200 mg ropivacaine (0.25%) according to a standardized regimen. The injection areas included deep tissues and superficial tissues, encompassing the superficial fascia and subcutaneous tissues. Postoperatively, patients undergoing total hip arthroplasty (THA) continued to receive diclofenac 75 mg twice daily, while patients undergoing total knee arthroplasty (TKA) were given diclofenac 75 mg twice daily and oxycodone 10 mg every 12 h. An intramuscular injection of morphine (10 mg) was administered when visual analogue scale (VAS) pain score exceeded 5. No non-protocolized pain medication was administered.

Patients received a standardized thromboembolic prophylaxis protocol, which consisted of a subcutaneous injection of 2000 IU enoxaparin at 8 h postoperatively and then once a day (4000 IU); in addition, 10 mg rivaroxaban was prescribed for 10 days after discharge. Metoclopramide was administrated intramuscularly if the patients experienced postoperative nausea and vomiting (PONV).

### Outcomes

The primary outcome was the FBG and ΔFBG (FBG_pod_ − FBG_pre_) on postoperative days (POD) 0–3. Secondary outcomes were postoperative PBG and ΔPBG (PBG_pod_ − PBG_pre_) levels on PODs 1–3. The length of stay, VAS pain scores on POD 1–3 were recorded. We also identified the risk factors for FBG ≥ 140 mg/dL and PBG ≥ 180 mg/dL. FBG was measured before breakfast. On operation day, the FBG was measured two times: immediately postoperatively and immediately upon returning to the ward. PBG was measured at 2 h after 3 meals. All BG level were measured using fingertip rapid blood glucose testing. Postoperative complications were recorded within 90-day follow-up.

### Statistical analysis

Based on previous studies, we estimated a mean 23 mg/dl (standard deviation, SD 27) of BG levels among groups during PODs 0 to 3 [27]. The sample size was calculated using PASS 2021 software (NCSS, LLC, Kaysville, UT) with a one-way analysis of variance designed for RCTs based on the results of previous studies. To detect a clinically meaningful effect of dexamethasone on postoperative FBG compared to placebo, a sample size of 36 participants was determined to achieve 90% power, assuming a type I error rate of 5%. Accounting for an anticipated attrition rate of 20%, we planned to recruit 135 patients.

Statistical analysis was performed by entering raw data into MS Excel for computation with IBM SPSS version 24 software (IBM, Armonk, NY). Categorical data are presented as absolute and relative frequencies. Continuous data are expressed as mean (SD). One-way analysis of variance and LSD post-hoc test was performed to compare continuous variables. Chi-squared test or Fisher's Exact test was used to analyze categorical data. Given that FBG ≥ 140 mg/dl and PBG ≥ 180 mg/dl are identified as risk factors for PJI [14–19], we performed multivariate analysis using logistical regression with backward stepwise conditional entry of the variables (*P* = 0.05 for entry; *P* < 0.10 for removal). The plausibly clinically relevant variables (age, gender, BMI, procedure, HbA1c, dexamethasone administration) were included. For elucidating the threshold of preoperative HbA1c level inducing hyperglycemia after TJA, a receiver operating characteristic curve with the Youden index was constructed. *P* < 0.05 indicated statistical significance.

## Results

### Study population

**Fig. 1:**
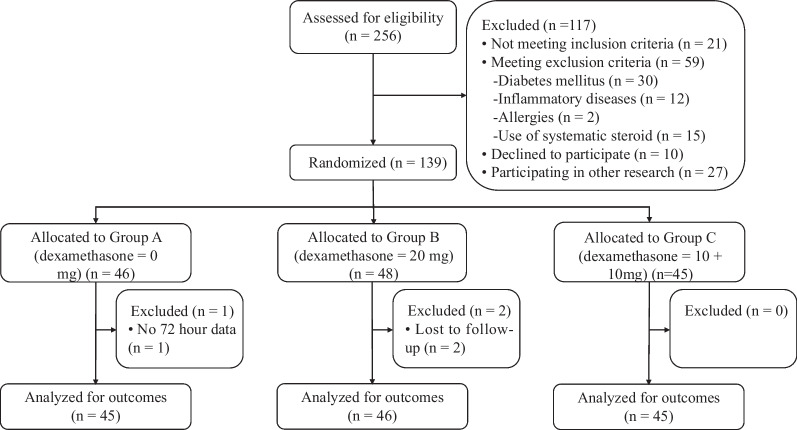
Participant flow diagram of the study design

Between May 3rd, 2023 and October 11th, 256 patients undergoing elective, unilateral, primary TJA for end-stage osteoarthritis were assessed for eligibility, of whom 136 were finally included in the study. Patients were randomized into the placebo group (Group A, n = 45), Group B (n = 46) and Group C (n = 45), respectively (Fig. 1). The baseline characteristics are presented in Table 1. No significant differences were observed among the groups.

**Table 1:** Baseline demographic and clinical characteristics

Characteristics	Group A (DEX = 0 mg)	Group B (DEX = 20 mg)	Group C (DEX = 10 + 10 mg)	*P* value
Demographic characteristics				
Age (year old)	58.9 (9.8)	59.0 (11.1)	61.0 (12.7)	0.602
Gender (M/W)	12/33	19/27	16/29	0.335
Height (m)	1.59 (0.07)	1.61 (0.08)	1.58 (0.08)	0.264
Weight (kg)	64.1 (10.4)	66.8 (13.4)	65.9 (10.3)	0.523
BMI (kg/m^2^)	25.0 (3.7)	25.6 (3.8)	26.2 (3.4)	0.334
Procedure (hip/knee)	21/24	23/23	22/23	0.949
Operated side (L/R)	19/26	23/23	22/23	0.725
Operation time (min)				
THA	81.8 (24.6)	81.2 (41.2)	90.2 (30.3)	0.600
TKA	90.0 (27.5)	92.7 (14.2)	81.3 (24.7)	0.217
ASA (I/II/III)	0/36/9	0/35/11	0/32/13	0.615
Laboratory examination				
HbA1c (%)	5.7 (0.4)	5.7 (0.6)	5.6 (0.4)	0.706
Preoperative FBG (mg/dL)	94.9 (12.7)	97.8 (28.2)	97.8 (12.9)	0.753
WBC count (10^9^/L)	7.1 (1.7)	7.2 (2.0)	6.5 (2.0)	0.184
ESR (mm/h)	20.4 (15.0)	18.9 (14.8)	27.1 (20.1)	0.058
IL-6(pg/mL)	3.5 (2.8)	2.7 (1.5)	3.1 (1.6)	0.203
CRP (mg/L)	3.4 (1.6)	3.4 (2.7)	3.8 (2.6)	0.591
Hemoglobin (g/L)	125.6 (12.5)	127.3 (16.9)	128.3 (13.8)	0.666
Albumin (g/L)	43.1 (3.7)	42.9 (3.1)	43.7 (3.8)	0.552
Preoperative VAS				
Dynamic	4.6 (0.7)	4.7 (0.6)	4.8 (0.9)	0.220
At rest	2.5 (0.6)	2.7 (0.6)	2.6 (0.8)	0.249
Preoperative SF-12 score				
PCS	46.5 (3.5)	45.6 (4.0)	45.9 (3.5)	0.495
MCS	50.4 (4.5)	49.7 (4.7)	49.6 (3.5)	0.643
Preoperative WOMAC score				
Pain	7.4 (2.7)	6.8 (2.0)	7.1 (2.7)	0.573
Stiffness	2.9 (1.7)	3.2 (1.7)	3.3 (1.9)	0.576
Function	36.1 (5.9)	36.4 (6.1)	37.9 (6.0)	0.304
In total	46.4 (7.3)	46.4 (6.6)	48.2 (6.8)	0.323

DEX, dexamethasone; M, men; W, women; BMI, body mass index; L, left; R, right; THA, total hip arthroplasty; TKA, total knee arthroplasty; ASA, American Society of Anesthesiologists; FBG, fasting blood glucose; WBC, white blood cell; ESR, erythrocyte sedimentation rate; IL-6, interleukin-6; CRP, C-reactive protein; VAS, visual analogue scale; SF-12, 12-item short form survey; PCS, Physical Component Summary; MCS, Mental Component Summary; WOMAC, the Western Ontario and McMaster Universities Osteoarthritis Index. Data are mean (standard deviation) unless stated otherwise

### Primary outcomes

**Fig. 2:**
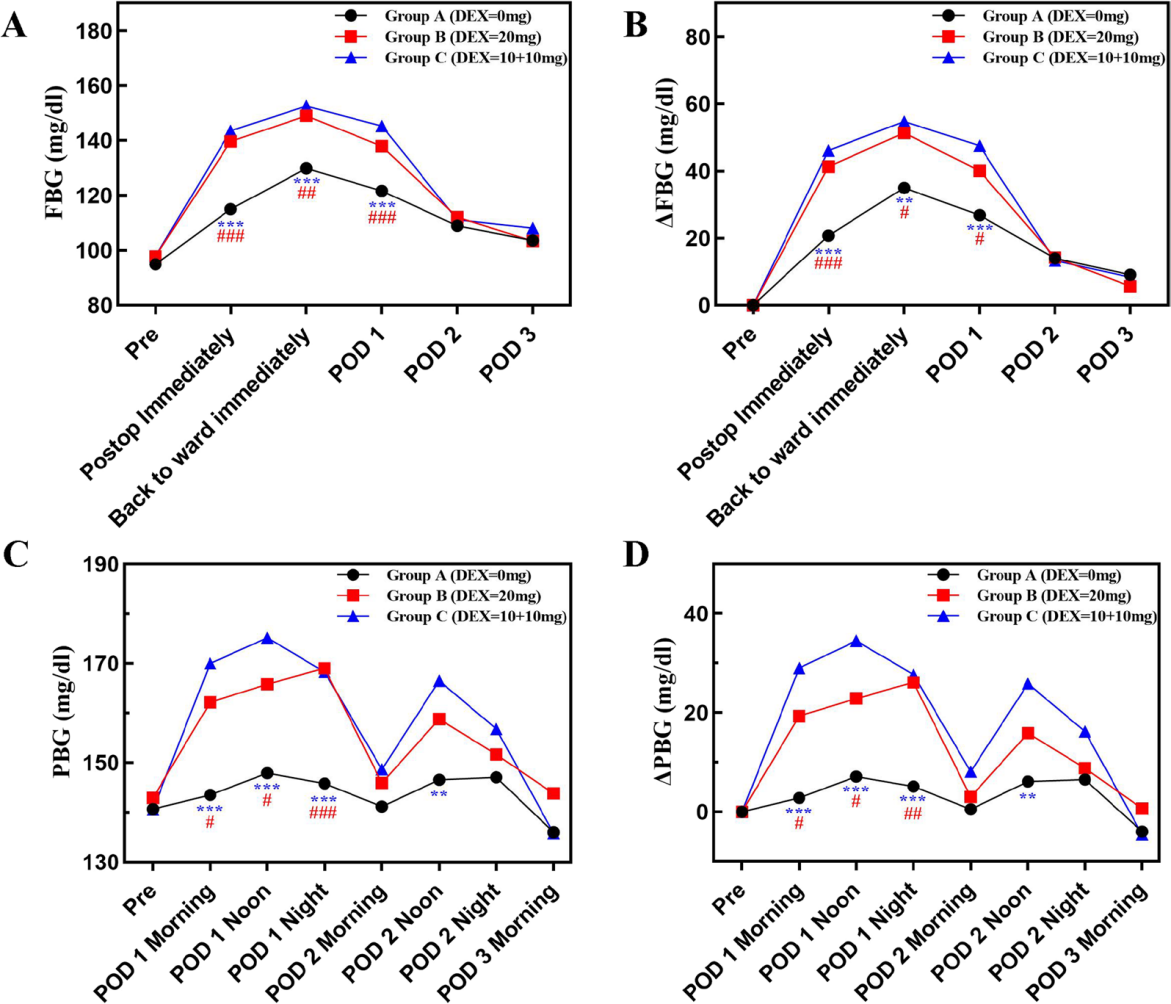
Postoperative **A** FBG, **B** ΔFBG, **C** PBG and **D** ΔPBG levels are according to the administration of intravenous dexamethasone after TJA. FBG, fasting blood glucose; TJA, total joint arthroplasty; Pre, preoperative; Postop, postoperative; POD, postoperative day; PBG, postprandial blood glucose. #*P1* (Group A vs Group B) < 0.05; ##*P1* < 0.01; *P1* < 0.001; *P2 (Group A vs Group C) < 0.05; **P2 < 0.01; ***P2 < 0.001

Compared to patients in Group A, patients in Groups B and C showed a transient increase in FBG on PODs 0 and 1 but no differences were discovered on PODs 2 and 3 among the three groups (Fig. 2A, B and Table 2). However, there were no differences in FBG between Groups B and C at any time interval (Table 2). When examining ΔFBG, Groups B and C demonstrated a similar higher change on PODs 0 and 1 compared to Group A, and there were also no differences detected between Groups B and C during hospital stay (Table 2).

**Table 2:** Postoperative outcomes after TJA

Characteristics	Group A (DEX = 0 mg)	Group B (DEX = 20 mg)	Group C (DEX = 10 + 10 mg)	*P* value	*P1* (Group A vs Group B)	*P2* (Group A vs Group C)	*P3* (Group B vs Group C)
FBG (mg/dl)							
POD 0 postop immediately	115.0 (25.6)	139.6 (26.4)	143.6 (26.3)	< 0.001*	< 0.001*	< 0.001*	0.486
POD 0 back to ward immediately	129.9 (31.2)	149.1 (32.0)	152.6 (21.8)	< 0.001*	0.002*	< 0.001*	0.562
POD 1	121.7 (21.6)	137.9 (22.8)	145.3 (26.2)	< 0.001*	0.001*	< 0.001*	0.136
POD 2	108.9 (17.5)	112.0 (16.9)	111.1 (16.3)	0.676	0.388	0.550	0.793
POD 3	103.6 (10.6)	103.4 (14.1)	108.1 (12.5)	0.129	0.939	0.089	0.072
△FBG (mg/dl)							
POD 0 postop immediately	20.7 (26.5)	41.3 (34.3)	46.1 (25.0)	< 0.001*	0.001*	< 0.001*	0.451
POD 0 back to ward immediately	35.0 (31.0)	51.3 (39.4)	54.8 (21.8)	0.008*	0.015*	0.004*	0.601
POD 1	26.8 (21.7)	40.1 (26.6)	47.5 (30.0)	0.001*	0.017*	< 0.001*	0.182
POD 2	14.0 (21.6)	14.2 (29.0)	13.3 (27.2)	0.983	0.970	0.891	0.861
POD 3	9.0 (13.6)	5.6 (26.4)	8.3 (21.5)	0.568	0.462	0.775	0.304
PBG (mg/dl)							
POD 1 Morning	143.5 (33.0)	162.2 (34.5)	170.0 (34.5)	0.002*	0.011*	< 0.001*	0.310
POD 1 Noon	147.9 (23.0)	165.8 (40.1)	175.2 (38.0)	0.001*	0.035*	< 0.001*	0.598
POD 1 night	145.8 (26.6)	169.0 (36.9)	168.3 (31.9)	0.001*	0.001*	0.001*	0.914
POD 2 Morning	141.2 (23.6)	145.9 (33.8)	148.7 (28.2)	0.481	0.453	0.231	0.649
POD 2 Noon	146.6 (20.3)	158.8 (41.2)	166.5 (35.8)	0.025*	0.230	0.006*	0.716
POD 2 night	147.1 (25.3)	151.7 (32.8)	156.8 (28.6)	0.303	0.457	0.123	0.414
POD 3 Morning	136.0 (28.5)	143.8 (35.2)	135.8 (32.8)	0.414	0.260	0.977	0.245
△PBG (mg/dl)							
POD 1 Morning	2.8 (32.4)	19.3 (34.2)	29.0 (35.0)	0.002*	0.023*	< 0.001*	0.182
POD 1 Noon	7.1 (22.8)	22.9 (39.5)	34.5 (38.2)	0.001*	0.034*	< 0.001*	0.115
POD 1 night	5.1 (26.7)	26.1 (36.5)	27.7 (32.0)	0.001*	0.002*	0.001*	0.822
POD 2 Morning	0.5 (23.3)	3.0 (34.1)	8.1 (27.7)	0.452	0.683	0.218	0.404
POD 2 Noon	6.1 (20.3)	15.9 (40.6)	25.9 (35.1)	0.024*	0.402	0.005*	0.510
POD 2 night	6.5 (25.7)	8.8 (32.6)	16.2 (28.8)	0.275	0.715	0.127	0.236
POD 3 Morning	− 4.0 (29.0)	0.7 (37.0)	− 4.6 (32.6)	0.710	0.505	0.936	0.452
Postoperative BG (mg/dl)							
Average FBG	116.1 (15.3)	127.9 (15.6)	132.0 (12.0)	< 0.001*	< 0.001*	< 0.001*	0.174
Average PBG	145.3 (18.5)	156.1 (28.4)	159.9 (21.8)	0.010*	0.029*	0.003*	0.437
Maximum BG (mg/dl)							
FBG (mg/dl)	141.6 (27.8)	160.6 (26.9)	166.3 (24.5)	< 0.001*	0.001*	< 0.001*	0.301
PBG (mg/dl)	173.8 (24.1)	189.1 (38.8)	201.0 (34.1)	0.001*	0.028*	< 0.001*	0.086
Postoperative Hyperglycemia							
FBG > 140 mg/dl (%)	5/40 (11.1%)	12/34 (26.1%)	11/34 (26.7%)	0.155	0.354	0.504	1.000
PBG > 180 mg/dl (%)	3/42 (6.7%)	5/41 (10.9%)	7/38 (15.6%)	0.404	1.000	0.942	1.000

TJA, total joint arthroplasty; DEX, dexamethasone; FBG, fasting blood glucose; PBG: postprandial blood glucose; BG, blood glucose; POD, postoperative day; postop: postoperative. *P < 0.05. Data are mean (standard deviation) unless stated otherwise

Additionally, patients in Groups B and C showed higher mean and maximum FBG levels than Group A within 72 h after surgery. Nonetheless, there were no differences among the three groups when comparing the proportion of individuals with mean FBG ≥ 140 mg/dl (Table 2).

### Secondary outcomes

On POD 1, the PBG and ΔPBG levels were higher in Groups B and C than in Group A, with a similar tendency between Groups B and C (Fig. 2C, D and Table 2). At POD 2 noon, a statistical difference was observed between Groups A and C in PBG and ΔPBG, while no difference was found on Group A and Group B or Group B and Group C. Meanwhile, The PBG and ΔPBG were similar among the three groups thereafter.

During hospital stay, Groups B and C trended towards slightly higher average and maximum PBG compared to that for Group A. Still, no differences were found in patients with postoperative PBG ≥ 180 mg/dl among the three groups (Table 2).

### Other outcomes

The dynamic pain scores on PODs 1, 2, and 3 were significantly lower for Groups B [4.80 (0.72), *P* < 0.001; 4.24 (0.57), *P* = 0.001; 3.39 (0.54), *P* < 0.001] and C [4.93 (0.25), *P* < 0.001; 3.80 (0.73), *P* < 0.001; 2.93 (0.65), *P* < 0.001] compared to Group A [5.36 (0.61); 4.73 (0.75); 3.93 (0.69)]. Such differences were also detected between Groups B and C on PODs 2 and 3 (*P* = 0.003; *P* = 0.001, respectively). There were no differences in length of stay, postoperative complications, 90-day readmission and 90-day mortality among the three groups (Table 3).

**Table 3:** Postoperative adverse events

Characteristics	Group A (DEX = 0 mg)	Group B (DEX = 20 mg)	Group C (DEX = 10 + 10 mg)	*P* value
Length of stay	3.7 (0.9)	3.7 (0.9)	3.5 (0.7)	0.473
Wound swelling	2/45	3/46	3/45	0.882
Wound ooze	2/45	2/46	2/45	1.000
Dizziness	4/45	3/46	2/45	0.700
Gastrointestinal hemorrhage	1/45	0/46	0/45	0.361
Superficial wound infection	0/45	0/46	0/45	–
Deep wound infection	0/45	0/46	0/45	–
PJI	0/45	0/46	0/45	–
DVT	0/45	0/46	0/45	–
90-day readmission	0/45	0/46	0/45	–
90-day mortality	0/45	0/46	0/45	–

DEX, dexamethasone; PJI, prosthetic joint infections; DVT, deep venous thrombosis

### Risk factors for postoperative hyperglycemia

**Fig. 3:**
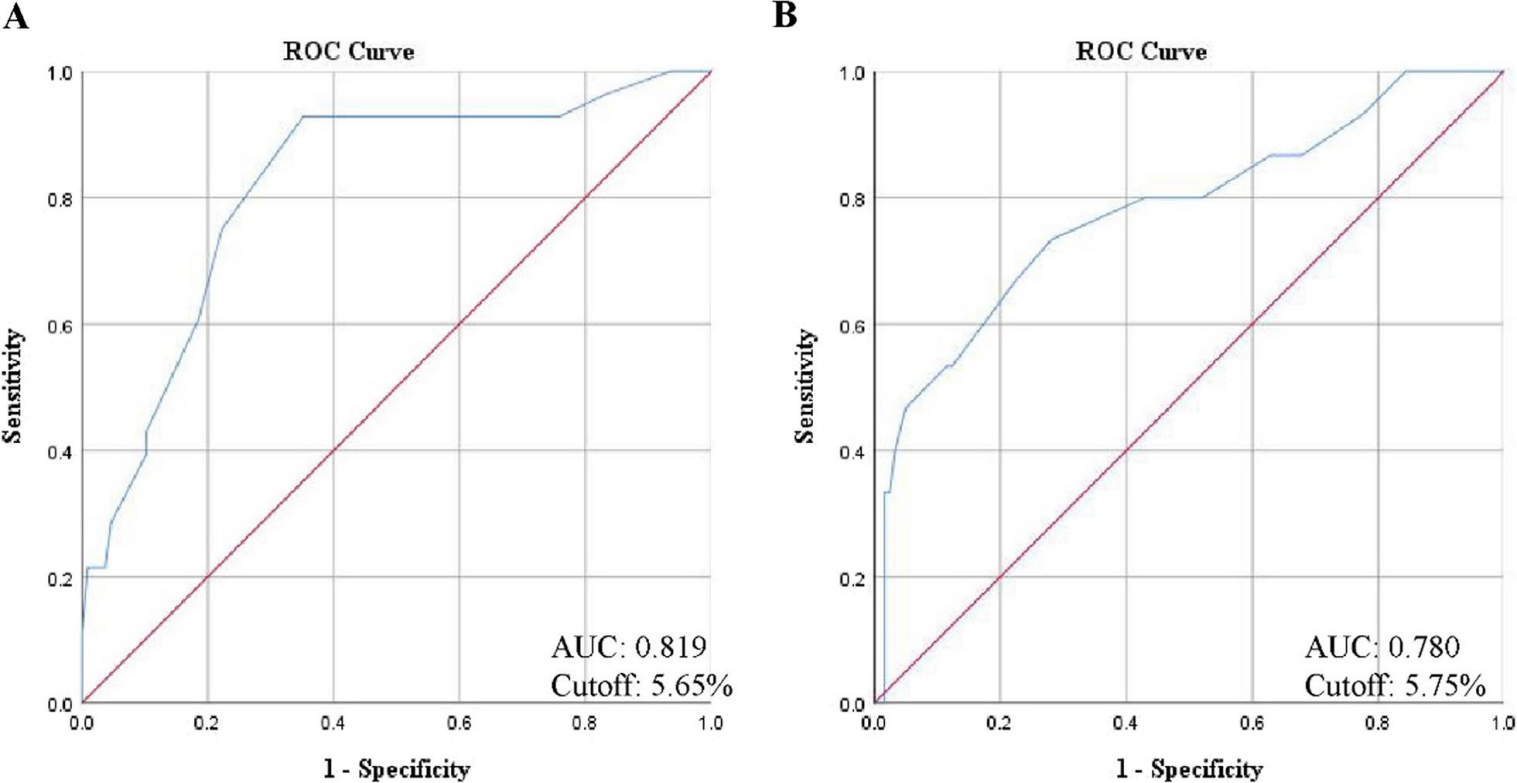
Receiver operating characteristic curve to determine the preoperative HbA1c level threshold. The threshold indicates the cutoff which increases the risk for the occurrence of FBG **A** ≥ 140 mg/dl or PBG **B** ≥ 180 mg/dl in patients without diabetes after TJA. FBG, fasting blood glucose; PBG, postprandial blood glucose; TJA, total joint arthroplasty; ROC, receiver operating characteristic curve; AUC, area under the curve

The risk factors for FBG ≥ 140 mg/dl were identified. In the multivariate analysis, binary logistic regression was employed to determine the odds ratio (OR) of different regimens of intravenous dexamethasone and other factors for the occurrence of FBG elevation. The ORs of intravenous dexamethasone increasing the postoperative FBG level to ≥ 140 mg/dl were 3.601 (0.883 to 14.692) (*P* = 0.074) and 3.612 (0.943 to 13.827) (*P* = 0.061), respectively (Table 4), indicating that compared to placebo, both dexamethasone regimens did not increase the risk of postoperative FBG level exceeding 140 mg/dl. However, the elevated preoperative HbA1c levels were associated with high postoperative FBG levels (OR = 12.738 (3.648 to 44.475), *P* < 0.001). The threshold for preoperative HbA1c level, associated with an increased risk of FBG ≥ 140 mg/dl occurrence, was determined to be 5.65% (Fig. 3A). The area under the curve was calculated to be 0.819.

**Table 4:** Logistic regression of risk factors for postoperative FBG level ≥ 140 mg/dl during total hospitalization

Variable	Univariate analysis		Multivariate analysis		VIF
	OR (95%CI)	*P* value	OR (95%CI)	*P* value	
Age (year old)	1.020 (0.982 to 1.059)	0.312			
Sex	0.773 (0.328 to 1.821)	0.556			
BMI (kg/m^2^)	1.159 (1.030 to 1.304)	0.015*	1.089 (0.952 to 1.245)	0.214	1.089
Procedure (hip/knee)	1.604 (0.687 to 3.741)	0.274			
HbA1c (%)	11.727 (3.871 to 35.527)	< 0.001*	12.738 (3.648 to 44.475)	< 0.001*	1.131
Preoperative DEX					
20 mg	2.824 (0.904 to 8.820)	0.074	3.601 (0.883 to 14.692)	0.074	1.039
10 + 10 mg	2.588 (0.818 to 8.188)	0.106	3.612 (0.943 to 13.827)	0.061	1.039

FBG, fasting blood glucose; DEX, dexamethasone; OR, odds ratio; CI, confidence interval

*P < 0.05

Similarly, we also conducted the multivariate analysis to demonstrate the risk factors for the elevation of PBG ≥ 180 mg/dl. Administering dexamethasone also did not increase the risk of the occurrence of postoperative PBG ≥ 180 mg/dl (OR = 1.535 (0.248 to 9.516), *P* = 0.645; OR = 4.213 (0.842 to 21.086), *P* = 0.080) (Table 5). Moreover, the elevated preoperative HbA1c levels were associated with high postoperative PBG levels (OR = 13.253 (3.612 to 48.625), *P* < 0.001). The threshold for preoperative HbA1c level, related with an increased risk of occurrence of PBG ≥ 180 mg/dl, was 5.75% (Fig. 3B). The area under the curve was 0.780.

**Table 5:** Logistic regression of risk factors for postoperative PBG level ≥ 180 mg/dl during total hospitalization

Variable	Univariate analysis		Multivariate analysis		VIF
	OR (95% CI)	*P* value	OR (95% CI)	*P* value	
Age (year old)	1.010 (0.963 to 1.060)	0.676			
Sex	1.063 (0.341 to 3.314)	0.916			
BMI (kg/m^2^)	1.126 (0.972 to 1.303)	0.113			
Procedure (hip/knee)	0.806 (0.275 to 2.361)	0.693			
HbA1c (%)	7.579 (2.747 to 20.911)	< 0.001*	13.253 (3.612 to 48.625)	< 0.001*	1.131
Preoperative DEX					
20 mg	1.707 (0.383 to 7.611)	0.483	1.535 (0.248 to 9.516)	0.645	1.039
10 + 10 mg	2.579 (0.622 to 10.690)	0.192	4.213 (0.842 to 21.086)	0.080	1.039

PBG, postprandial blood glucose; DEX, dexamethasone; OR, odds ratio; CI, confidence interval

*P < 0.05

## Discussion

In this prospective, randomized, controlled and double-blind trial, we compared the postoperative glycemic control of a single high-dose of dexamethasone with a split-dose dexamethasone regimen in patients without diabetes undergoing primary elective TJA. We found that there were transient increases in FBG and PBG on PODs 0 and 1 when dexamethasone was used no matter the split-dose or single high-dose regimens. However, neither the split nor single, high-dose of dexamethasone administration increased the risk for postoperative FBG ≥ 140 mg/dl or PBG ≥ 180 mg/dl.

Intravenous dexamethasone has been used to mitigate not only PONV but also pain control following TJA [28, 29]. Nevertheless, there remains concerns that the use of intravenous dexamethasone may elevate BG levels and potentially contribute to an increase in wound complications or PJI [25]. Some published literature has discussed the association between dexamethasone and BG. Allen et al. documented a temporary increase in BG levels on POD 0 in 825 patients with diabetes who received 8 mg of dexamethasone [22]. Godshaw et al. illustrated that the administration of dexamethasone at doses of 6 or 12 mg had no impact on postoperative BG levels in 657 patients with diabetes undergoing TJA [24]. Nurok et al. demonstrated that postoperative hyperglycemia in a cohort comprising 625 patients with and without diabetes was not correlated with the administration of dexamethasone at a median dose of 4 mg [23]. Nonetheless, a systematic review indicated that the administration of intravenous dexamethasone increased BG levels within 24 h after surgery [12]. In our study, we noted that split-dose and single high-dose dexamethasone both led to a transient elevation in FBG on PODs 0 and 1. However, no differences were detected beyond POD 1, consistent with findings from previous studies. Furthermore, our study revealed that patients administered dexamethasone experienced a greater fluctuation in FBG compared to those receiving a placebo. However, there was no significant difference observed between the split-dose and single high-dose groups, and the elevated levels swiftly returned to normal. This observation may be attributed to the heightened sensitivity of patients without diabetes to dexamethasone-induced hyperglycemia [20]. Hence, in the dexamethasone groups, the rise in BG levels was more pronounced compared to placebo, leading to a larger disparity from preoperative levels and more significant overall fluctuations.

Recently, the American Society of Regional Anesthesia and Pain Medicine (ASRA) stated that administration of multiple doses of perioperative intravenous dexamethasone results in reduced pain, decreased opioid consumption, and less nausea/vomiting compared to a single dose of perioperative intravenous dexamethasone [1]. However, whether the use of multiple doses of dexamethasone has a more substantial impact on BG remains unclear. And there lacks reports on the association of different doses of dexamethasone and postoperative hyperglycemia in patients without diabetes undergoing TJA. Arraut et al. observed that in 523 patients with diabetes receiving THA and 953 patients with diabetes receiving TKA, 2 doses of 10 mg dexamethasone was associated with increased postoperative 24-h and 60-h BG levels compared to 1-dose cohort, nonetheless, the study was retrospective [21]. Lei et al. found no differences in FBG between patients receiving a perioperative 2 doses of 10 mg dexamethasone and a preoperative 20 mg dexamethasone undergoing TJA, however, they included patients with and without diabetes, which compromised the accuracy of the conclusions [11]. Park et al. investigated the effects of administering 2 doses of 5 mg dexamethasone in 427 patients without diabetes undergoing TKA and observed a transient increase in BG levels. However, this increase was not associated with levels exceeding 200 mg/dl [25]. In our study, as we mentioned before, the FBG was transient increased on PODs 0 and 1 in both split-dose and single high-dose groups. The PBG and ΔPBG levels were also higher in single high-dose group on POD 1 compared to placebo. Although differences were found between split-dose group and placebo on POD 2 noon, yet the both average PBGs were less than 180 mg/dl, which did not increase the rates of wound complication and infection. Moreover, no differences were found among the three groups in the subsequent time points.

Postoperative hyperglycemia is an important indicator for adverse events in surgical patients, irrespective of diabetes status [14]. Moreover, postoperative hyperglycemia may increase the risk of adverse events more significantly in patients without diabetes, further emphasizing its clinical relevance [15]. Additionally, differentiating between FBG and PBG is crucial. Given that the different indicators represented by FBG and PBG. Fasting hyperglycemia is a sign of insulin resistance, inadequate insulin production, or a combination of both [30]. Postprandial hyperglycemia, on the other hand, is indicative of insulin resistance and poor glucose tolerance [31]. Moreover, the threshold values for postoperative hyperglycemia in patients without diabetes vary between FBG and PBG, and some institutions may only monitor FBG data while others may only monitor random BG data in patients undergoing TJA. Mraovic et al. conducted a perioperative hyperglycemia examination involving 1,948 patients, including 101 individuals with PJI. The study observed that patients with PJI exhibited higher FBG levels on POD 1 compared to those without PJI. Interestingly, their findings also indicated that in cases where postoperative FBG levels increased (> 140 mg/dl), patients without diabetes were three times more likely to develop infections than patients with diabetes (*P* = 0.001) [17]. Kheir et al. reported that the association between postoperative FBG levels on PODs 0, 1, and 2 and the occurrence of PJI exhibited a linear trend. They further identified that the optimal FBG threshold, aiming to minimize the likelihood of PJI, was determined to be 137 mg/dl [16]. In contrast, Kremers et al. demonstrated that elevated random BG levels exceeding 180 mg/dL on PODs 1 to 7 were associated with an increased risk of PJI in patients undergoing TJA [18]. In a study by Kwon et al., which included 11,633 patients undergoing elective colorectal and bariatric surgery, it was observed that postoperative random BG levels surpassing 180 mg/dl on PODs 0, 1, and 2 were linked to postoperative adverse events [14]. It is challenging to definitively determine the relative importance of FBG and PBG in detecting PJI or other postoperative adverse events. However, based on current literature, we suggest that identifying risk factors for FBG levels ≥ 140 mg/dl and PBG levels ≥ 180 mg/dl is crucial in preventing PJI. In our study, the increase in BG induced by dexamethasone was statistically significant only on PODs 0 and 1. However, this transient elevation did not exceed the threshold associated with complications for postoperative FBG or PBG. Therefore, dexamethasone-induced BG elevation may show no clinical significance. Considering the actual analgesic and antiemetic effects of dexamethasone, we believe that its efficacy may still outweigh its adverse effects.

Postoperative hyperglycemia may be the most important risk factor for surgical site infection, PJI or other complications [32, 33]. Therefore, identifying risk factors for postoperative hyperglycemia is beneficial for early prediction of its occurrence. Godshaw et al. indicated that elevated BG levels and increased rates of PJI were associated with the elevation of HbA1c rather than the use of dexamethasone [34]. Moorthy et al. stated that in patients without diabetes, those who experienced postoperative hyperglycemia tended to be female, older, and obese [35]. Hence, in multivariate analysis of risk factors of postoperative hyperglycemia, we included the following indicators: dexamethasone administration, HbA1c which represents BG levels of the last 90 days, age, gender, procedure and BMI. We have reported for the first time the risk factors associated with postoperative elevation of FBG and PBG, offering a more detailed analysis compared to previous studies. Interestingly, we found that neither regimen of dexamethasone administration was a risk factor for FBG or PBG elevation, which is in alignment with previous studies. The sole risk factor for postoperative FBG ≥ 140 mg/dl and PBG ≥ 180 mg/dl was preoperative elevated HbA1c. Additionally, patients with a preoperative HbA1c level above 5.65% and 5.75% may encounter postoperative elevated FBG and PBG respectively, irrespective of dexamethasone dose and injections.

This study also has several limitations. We only observed changes in BG levels until POD 3 and postoperative complications until POD 90. The long-term effects of dexamethasone on BG and its potential association with long term adverse events remain unexplored, and we will continue to follow up with our patients, aiming to report on the long-term effects of different dexamethasone regimens in future studies. Secondly, our patients may include individuals with prediabetes, and we did not differentiate this subgroup of patients. It remains unclear whether dexamethasone has different effects on BG of these patients while this lack of distinction may introduce bias into the study. Therefore, we will conduct further investigations on the effects of dexamethasone on BG in patients with prediabetes, aiming to elucidate its impact on BG in different populations.

## Conclusions

Split-dose or single high-dose of intravenous dexamethasone administration perioperatively had transient effects on increasing BG levels within POD 1 in patients without diabetes who underwent TJA. However, no differences were found between the split-dose and a single high-dose regimen. And no differences were detected on BG levels beyond POD 1. Additionally, both dexamethasone regimens did not increase the risk of FBG ≥ 140 mg/dl and PBG ≥ 180 mg/dl. The threshold for preoperative HbA1c levels associated with an increased risk of postoperative FBG ≥ 140 mg/dl and PBG ≥ 180 mg/dl was found to be 5.65% and 5.85%, respectively, regardless of the dexamethasone regimen.

## Data Availability

The datasets used and/or analyzed during the current study are available from the corresponding author on reasonable request.

## Abbreviations

TJA: Total joint arthroplasty
BG: Blood glucose
FBG: Fasting blood glucose
PBG: Postprandial blood glucose
POD: Postoperative day
PJI: Periprosthetic joint infection
THA: Total hip arthroplasty
TKA: Total knee arthroplasty
VAS: Visual analogue scale
PONV: Postoperative nausea and vomiting
SD: Standard deviation
OR: Odds ratio
ASRA: American Society of Regional Anesthesia and Pain Medicine
Pre: Preoperative
Postop: Postoperative
ROC: Receiver operating characteristic curve
AUC: Area under the curve
DEX: Dexamethasone
M: Men
W: Women
BMI: Body mass index
ASA: American Society of Anesthesiologists
WBC: White blood cell
ESR: Erythrocyte sedimentation rate
IL-6: Interleukin-6
CRP: C-reactive protein
SF-12: 12-Item short form survey
PCS: Physical Component Summary
MCS: Mental Component Summary
WOMAC: The Western Ontario and McMaster Universities Osteoarthritis Index
DVT: Deep venous thrombosis
CI: Confidence interval
